# The interplay of ethics and genetic technologies in balancing the social valuation of the human genome in UNESCO declarations

**DOI:** 10.1038/s41431-024-01549-3

**Published:** 2024-02-15

**Authors:** Hristina Gaydarska, Kayo Takashima, Shibly Shahrier, Aviad Raz, Jusaku Minari

**Affiliations:** 1 Uehiro Research Division for iPS Cell Ethics, Center for iPS Cell Research and Application (CiRA), Kyoto University, Kyoto, Japan; 2 Teesside University International Business School, Teesside University, Tees Valley, UK; 3 Department of Sociology and Anthropology, Ben-Gurion University of the Negev, Beersheba, Israel

**Keywords:** Economics, Ethics

## Abstract

This study investigates changes in the social valuation of the human genome over the more than 30 years since the establishment of the Human Genome Project. It offers a descriptive sociological analysis of the three waves of this valuation, mainly by considering three key UNESCO declarations and a relevant report. These waves represent a shifting balance between collectivism and individualism, starting with a broadly constructed valuation of the human genome as common human heritage and moving toward a valuation of dynamic applications within various social and medical contexts (e.g., personalized genomic medicine and genome editing). We seek to broaden the analytical perspective by examining how the declarations’ ethical foci are framed within the context of rapidly evolving genetic technologies and their social applications. We conclude by discussing continuity and change in value balancing vis-à-vis changing genomic technologies.

## Introduction

Since the completion of the Human Genome Project (HGP), rapid advances in genomic research have shifted the landscape of the human genome, challenging some of the universal principles and values related to human rights. To explore which values related to the human genome have remained stable over time and which have changed, we focus on UNESCO’s three bioethics declarations—the 1997 Universal Declaration on the Human Genome and Human Rights (UDHGHR) [1], the 2003 International Declaration on Human Genetic Data (IDHGD) [2], and the 2005 Universal Declaration on Bioethics and Human Rights (UDBHR) [3]—and the 2015 Report of the International Bioethics Committee (IBC report) [4]. Notably, the UDHGHR and the IBC Report [1, 4] refer to the human genome as the “heritage of humanity,” arguing that it should be protected while being passed on to future generations and that technological and scientific advances must be considered in light of human rights. Such international initiatives can thus be regarded as preparation for and a response to the challenges facing the social valuation of the human genome, which are often driven by the coevolution of society and new genetic technologies.

While several international documents reflect a consensus on the right to benefit from genome research and its applications, UNESCO’s bioethics initiatives are special in three main ways. First, the consecutive UNESCO publications (1997, 2003, 2005, and 2015), which reflect technological developments and related ethical issues, present interconnected international norms. Second, previous studies related to these declarations [5–13] have identified key ethical, legal, and social issues, providing a solid base for expansion. Several studies have discussed the process of drafting, implementing [5–7], and changing emphases of the critical principles and norms in the UNESCO documents [8–13]. Third, with the evolution of genetic technologies and the emergence of various new approaches, the nature of the human genome’s social valuation has become increasingly subject to change [14, 15]. Given the expanding applications of the human genome [16], such consideration is needed, as reaching a consensus on these declarations often requires contending with legal and social debates over whether the human genome should be regarded as a “common good” [4, 17].

The example of water, another well-known “common good,” illustrates the diverse and changing nature of valuation. One approach to grasping water is to consider it a “global public good.” Water has fundamental value for everyone; therefore, each individual should have free access to it. This universal concept of water differs from its economic valuation as a private good. Another approach is that water’s valuation is contextual: it depends on timing, availability, interconnectivity, and quality, indicating that the value of water can vary due to different stakeholders’ competing perspectives and uses. However, many goods do not fall into either a public or private category [18]. In this regard, there is no international consensus on its valuation, but the perspective of its global common good is increasingly being necessitated [19]. Compared to water, a physical commodity, the digital commodification of the human genome includes more ambiguity regarding sustainable usage and sharing among stakeholders.

This paper aims to explore the links among genetic technologies, the corresponding social valuation of the human genome, and the ethical implications of such valuation. In particular, given that both the tangible (e.g., medical and technological) and intangible (e.g., social and ethical) values of the human genome have changed—as manifested through various ethical, legal, and social issues, such as health equity, economic cost, clinical value, value for patients, and affordability [18, 20, 21]—we explore the heterogeneity and dynamics of the social valuation of the human genome by distinguishing three phases or categories of social valuation: fundamental or universal, contextual, and negotiated or contested.

### The three waves of the UNESCO declarations and the International Bioethics Committee Report

The IBC is a core international forum for in-depth reflection on bioethics. As a representative institution among the UN agencies responsible for the promotion of scientific research, it is appropriate for UNESCO to fulfill the role of establishing an international legal framework for the protection of the human genome, considering both its individual and collective responsibilities [5]. However, from the outset, the intricacies of the declaration development process were evident because of the diversity of value systems and disparate social and cultural standings [6].

The three waves of the UNESCO declarations and the IBC report reveal the internal ethical logic underlying the evolution of the dominant explanation. This evolution in the IBC program begins from the first declaration, the UDHGHR (1997), with a general outline aiming to balance genomic research with human rights and social virtues. The second declaration, the IDHGD (2003), complements the first by offering more practical, biomedically oriented ethical guidelines for the use of human genetic data. The third declaration, the UDBHR (2005), provides an integrated view of bioethics in which genomics is one challenge among many. The IBC report (2015) includes reflections on ethical perspectives and key challenging areas [4].

The link between the first declaration and the HGP is well known, and there has been much discussion about how the declarations provide an ethical framework for particularly challenging genetic and related technologies, including human cloning, and avoid sensitive issues, such as embryo research, that invoke religious or cultural controversies. However, this outlook interprets the use of these technologies as disconnected ethical challenges and fails to consider their underlying patterns of technological change. Our theoretical contribution is to broaden the analytical perspective to examine how the ethical foci of the declarations and the IBC report are embedded in the context of evolving genetic technologies and social settings. We also apply this perspective to account for changes in the declarations’ ethical balancing. Such a sociological analysis raises new questions as to why these issues were defined as ethical concerns in those times and places [22, 23]. We use the term “valuation” to highlight the socially dynamic and contextually embedded interplay of genetic technologies and ethics, as it juxtaposes changes in the construction and sequencing of the human genome with the ethical challenges emphasized by the declarations.

This view is demarcated into three separate “waves.” The first focused on the universal governance frameworks needed to ensure that the ethical, legal, and social implications of the human genome—then presumed to be singular—were appropriately addressed. The second wave focused on pluralism and diversity as well as standards of practice. This coincided with a shift from the HGP, which derived primarily from 11 donors, to the International HapMap Project (2002–2016), which examined genetic variation (in single nucleotide polymorphisms [SNPs]) among many human populations. The third and current wave focuses on the contested valuation and reevaluation required to balance universal/fundamental and individual/practical facets, emphasizing social grouping in genomic research and the challenges of its ethical, legal, and social implications.

### The first wave: UNESCO’s Universal Valuation of the Human Genome Project

UNESCO’s UDHGHR was proposed by the IBC, a special body established in 1993, which was the only ethics body within the UN system that has no counterpart at the international level [5]. This proposal was adopted in 1997 at UNESCO’s 29th General Conference, although related discussions started 4 years earlier. The UDHGHR, which comprises 25 articles, emphasizes that the human genome “underlies the fundamental unity of all members of the human family, as well as the recognition of their inherent dignity and diversity” [1, Article 1]. The main principles related to human dignity highlight autonomy, equality, and solidarity as fundamental human rights and virtues [6]. The first paragraph of the declaration defines the human genome as a symbol of the “heritage of humanity,” and the document later raises human dignity as an objection to human cloning [1, Article 11]. The universal principles, as described in the UDHGHR, are closely related to the collective valuation of the human genome [24].

Progress in genetics during the 1990s, represented by the development of the HGP, provided the impetus for the first UNESCO declaration [25]. This declaration indicates UNESCO’s concern about balancing scientific progress with the protection of human rights in international contexts [6].

The UDHGHR echoes the HGP in constructing an image of a single unified valuation of the human genome, which has a tangible form of approximately three billion pairs of DNA and was developed with first-generation technology (e.g., Sanger sequencing). Similar with the declaration’s call for open access, the entire human genome database and technological tools are free and universally available [26]. The UDHGHR thus addresses the human genome’s intrinsic value and the multilateral consensus on its solidaric sharing, while avoiding discussions of its potential abuse and embryo research.

Because of its general nature, the declaration was criticized as an exercise in pragmatic ethics that reiterated existing human rights principles within the genetic context but did little to articulate how this declaration might be utilized “on the ground” [6].

### The second wave: UNESCO’s contextual valuation of human genetic data

UNESCO’s 2003 IDHGD, which comprises 27 articles, was a response to rapid technological developments in the genomics field. Discussions began in 2001, 2 years before its adoption, when UNESCO’s director general proposed that the IBC examine the possibility of drafting an international instrument on human genetic data. While the first declaration is “universal,” the second emphasizes internationalization (specifically the plurality of UNESCO’s member states) and is more specific regarding what constitutes human genetic data. It extends the general principles of equality, autonomy, justice, and solidarity to address practical, individual-focused ethical issues, such as consent, privacy, benefit sharing, harm, and non-discrimination. In addition, the second declaration replaced the UDHGHR’s general characterization of the human genome as the common heritage of “all members of the human family” with references to “human genetic data” and individual differences. Words such as “human being” and “mankind” were likewise replaced with terms such as “individuals,” “personal genomic data,” and “identifiable persons.”

Given the completion of the HGP and the subsequent availability of genetic data, the second UNESCO declaration specifically addressed the increased possibility of their medical applications and the potential fear of misuse [25, 27].

The IDHGD echoed the completion of the HGP and the shift to the International HapMap Project, which aimed to construct not a single universal human genome but an international map of common patterns of differences in human haplotypes. At this time, a new approach to genome-wide association studies was developed, enabling the rapid and meaningful cross-reading of SNPs, the most common genetic variation among people. This second wave addressed the human genome’s intrinsic and contextual medical value. Consequently, a specific goal of “non-discrimination and non-stigmatization” [2, Article 7] emerged during this individual-centered stage.

### The third wave: contested valuation of human genetics in sociocultural contexts

The UDBHR originated from UNESCO’s General Conference in 2004, which addressed the need to draft a “universal instrument on bioethics.” The final version includes 28 articles, and unlike the previous declarations, it emphasizes general principles related to bioethics. Specifically, the UDBHR refers to respect for social responsibilities, balancing contradictions, and assessing potential social discrimination and inclusion in genomic research. It also broadly applies these ethical issues to medicine, life sciences, and associated technologies, “taking into account their social, legal, and environmental dimensions” [3, Article 1].

The UDBHR addresses the proliferation of practice and the growing need for international ethical standards in biomedicine, as biomedical research projects extend beyond national borders, often without regulatory frameworks. This expansion of biomedical research and clinical practice, including genomics and stem cells, along with the basic right to access medicines, nutrition, and water, necessitated the adoption of an instrument for global minimum standards to be promoted in nations lacking such standards [9, 28]. Andorno suggested that the generality in the formulation of the principles is justified by the need to respect cultural diversity, as nations have different definitions of autonomy, justice, and solidarity [9].

The UDBHR broadened the bioethical outlook beyond individual rights in the genetic context by considering additional medical and research contexts. For example, UNESCO’s decision to identify “social responsibility for living conditions” as a bioethical principle considerably expanded the view of bioethics in a way that mirrors developments in the philosophy of human rights [9]. The article on social responsibility—which declares that both states and societies have a duty to promote health and social development, enhance access to healthcare, nutrition, and water, reduce poverty and illiteracy, and eliminate social exclusion [3, Article 14]—was approved by consensus after developing countries asserted its paramount importance [3]. In this manner, the UDBHR problematized the proclaimed “universal” status of presumably “common goods” (e.g., nutrition, water, healthcare, and the human genome), the control of and access to which produce systemic social inequalities. It also highlighted group rights, claiming that “groups of special vulnerability should be protected” [3, Article 8] and that “the importance of cultural diversity and pluralism should be given due regard” [3, Article 12]. According to Magnus, although the UDBHR could not ensure the application of the common principles as well as the shared values in practical terms, it strived to provide a more comprehensive understanding of such principles by issuing further guidance [12].

In 2015, UNESCO published the IBC report, which included updated reflections on the human genome and human rights, to address current challenges and developments in genomics [4]. Dialog on this report began in 2014, when the IBC revisited its earlier initiatives on the human genome and human rights. The report was created in response to the increasing attention paid to precision/personalized medicine, biobanks, and emerging techniques for engineering gametes and human genomes [4]. The document synthesized the previous declarations, reaffirming their main principles while focusing on several new phenomena related to those biotechnologies.

UNESCO is not an oversight body and has no particular mechanisms to implement or enforce its aspirational statements. However, by focusing on the core values that all countries share, the UNESCO declarations have contributed to establishing a minimum standard for conducting biomedical research. Enforcing these values in domestic policies and regulations depends on the goodwill of national signatories. While many of these principles are abstract and not explicitly defined, their interpretation by member states is crucial.

To recapitulate, UNESCO published four key international documents that encapsulate the complex shift of balancing, from a universal/collective stage (i.e., a general and single emphasis on “our” human genome as a cherished human heritage) to an individual-focused stage that emphasizes the plurality of international governance and the personalization of genetic data in medicine, and finally, to a stage that blends individualism and collectivism, focusing on social responsibility and group vulnerability. These shifts represent a transition in the human genome’s emphasis from upstream activities, such as scientific discoveries, to downstream activities for medical, economic, and other purposes. In addition, there has been a shift from valuing the human genome as common human heritage (i.e., heritage implies equating it to require preservation, such as a UNESCO World Heritage site) to valuing it for its dynamic applications and even alterations (e.g., personalized genomic medicine and genome editing). These shifts also reflect a change in focus from a conceptual risk to “human dignity” to the social risks faced by vulnerable groups experiencing health inequalities, as the new approach identifies more concrete medicolegal risks in the context of autonomy and genetic discrimination.

### Balancing values in a social context

Regarding the rebalancing of the human genome valuation, the preliminary stage of a collective, purportedly single, universal (“our human heritage”) genome highlights the human genome’s fundamental value as a natural asset that should not be cloned, as doing so would infringe on human dignity. The HGP promoted rapid collective data sharing through the Bermuda Principles (1996) [26], which benefited the global scientific community. However, even though the HGP enabled significant achievements in genome research, it did not result in significant and immediate medical uses to revolutionize the diagnosis and treatment of many illnesses [29].

As scientists shifted their attention from genetic unity to genetic diversity, the International HapMap Project revealed SNP variance in human populations. In addition, given that monogenic diseases account for only a small proportion of socially significant diseases, genetic approaches tend to focus on the polygenic causes of common and chronic diseases. This period also saw a transition from viewing DNA as a predetermined “book of life” to recognizing epigenetics [30].

Emphasizing the value of diverse population genetics and aiming to close the translational gap, several countries established disease-specific genomic biobanks during this period. Particularly in cancer genomics, the landmark Cancer Genome Atlas Program (2000–2013) achieved significant progress in cancer genetics classification, stratification, and prognosis. However, the expected applications of the HGP to pharmacogenomics and medicine have not yet been fully realized [31]. Furthermore, the gaps in public knowledge of genetics [32], the transformation of medicalization to biomedicalization [33], and the clinical applications of genomics began to widen at this time. With the promise of personalized genomic medicine during the second stage, the diversification and personalization of genomic data also highlighted data protection and privacy concerns that impinge on data sharing, as individuals could be identified through their SNPs.

The third and current stage is balancing values and reflects the promotion and diversification of genome analysis. The cost of whole genome sequencing has decreased 10,000-fold—from one billion to a few thousand dollars—which has changed the relevance of and access to genomic data. Thus, numerous new genetic technologies have emerged [34], each tailored to a particular purpose, highlighting the increasing challenges of individualized ethical matters [35]. With the personalization of genomic data, the European Union recognized the need to protect personal information and enacted the General Data Protection Regulation 2016/679 (GDPR). This current stage has also built on and extended previous progress in cancer diagnostics and treatment through initiatives such as the US Cancer Moonshot Program (originally launched in 2016), which has decreased screening detection gaps for various cancer types and increased access to treatment using stratification methods.

To address missing heritability [36], genetics-based approaches can now screen for multiple factors associated with common conditions by combining hundreds of rare genes from healthy and diseased populations that may be associated with the occurrence of common diseases.

Streams for stratified medicine, as represented in the polygenic risk score (PRS), increasingly raise ethical and legal concerns regarding multiple groupings (such as gender, ethnicity, and age), indicating yet another aspect of rebalancing individuals, social groups, and society at large. Genomic public health approaches target at-risk groups and populations and manifest a resurfacing of previous social groupings, potentially neglecting crucial systemic health disparities that reflect social inequalities [37]. However, genomic risk prediction may soon emerge as a common stratification tool for chronic diseases, such as coronary artery disease, type 2 diabetes, and inflammatory bowel disease [38]. When the PRS is applied to patients stratified by self-identified race or ethnicity, it may have a range of consequences, because the designation of ethnicity is imprecise, highly ambiguous, and far from an objective counterpart to race [39]. Since the language used to describe individuals or groups can be imprecise or even offensive, genetic approaches could avoid this by selecting words such as “multi-population” or “cross-population” over inaccurate words such as “trans-ethnic” [40].

Clinical use of the PRS could also exacerbate race-based health disparities and reinforce systemic biases in the use of self-reported race, ethnicity, and ancestry as biomarkers and risk factors for disease diagnoses [41]. While many complex traits and diseases differ in prevalence among racial or ethnic groups, this results from pronounced racial and ethnic health disparities rather than genetic differences. Race-based pharmacogenetic screening recommendations may likewise result in considerable practice variations and stereotyping, causing unknown clinical consequences and reinforcing preexisting beliefs about race as a biological construct [42]. It has been argued that the PRS poses similar ethical, legal, and social issues to individual genetic results and incidental findings [41]. However, the PRS has different implications from monogenic disease diagnostics in that it combines information across many genetic variants into a weighted sum, providing an imperfect prediction of future health status. Its statistical value is much more ambiguous than that of the clinical context of monogenic diseases and is, therefore, less actionable. Despite the current popularity of the PRS due to direct-to-consumer genetic testing services, no robust guidelines for conducting and interpreting these predictions have been issued.

In the case of population-based genomic research, including the US All of Us Research Program, indigenous and ethnic groups have recently contested the “illusion of inclusion” in issues of ownership, control, access, and use of their members’ common genomic data [43]. These initiatives, along with the social disparities and group vulnerabilities they manifest, demonstrate that the UDBHR and IBC report are more relevant than ever.

Recently, not only human genomes but also their modifications have been increasingly discussed in the global context [44]. Applications of such modifications include germline genome editing in humans. If we follow the first declaration’s original perspective, which regards the human genome as common human heritage that needs to be preserved, then, in principle, genome modifications must be considered cautiously. Primarily, alternative social approaches must be explored to save and care for future patients, rather than social adoption of genome editing. This includes the optimization and enhancement of health and social care (e.g., how to fairly and efficiently distribute public and private subsidies) and the encouragement of community and social understanding and engagement. The rationale for this claim stems from the uncontrollability, unpredictability, and irreversibility of germline genome editing in society, as predicting and managing the social impact on present and future generations remain challenging.

### Discussion: lessons and future directions

UNESCO’s concern with moral issues in science has provided this reflection with a window into changing genetic technologies and their moral implications in various social settings. As Julian Huxley, the first UNESCO director general, pointed out, guiding the development of science for the benefit of humanity implies “the quest for a restatement of morality … in harmony with modern knowledge” [45]. One concern repeated in all three UNESCO declarations and the IBC report is the need for public education and engagement in genomic research. Indeed, managing public access to, use of, and knowledge of genomic information is increasingly important [46, 47]. As this article demonstrates, while expert social valuation of the human genome may be presented as “universal,” it is contingent on technological changes and social circumstances. While experts cannot determine the value of the human genome for individuals, as this is a matter of construction, the public cannot determine broad social values without the agreement of experts [48]; thus, social valuation must always be negotiated to shape the nature of the global common good of human genomes.

Other scholars have offered normative–ethical criticism of the consistency and comprehensiveness of the UNESCO declarations [8, 9]. For example, applying these declarations to the dignity of the individual, family, community, and human species gives a particular valuation that supports conflicting consequences, thereby reducing its utility as an evaluative moral concept. The declarations present a plurality of potentially contradictory fundamental values, including autonomy, solidarity, equality, democracy, and respect for life. However, they lack ranking mechanisms or decision-making frameworks for managing such conflicts and offer minimal practical guidance for appropriately balancing values [10]. Through UNESCO initiatives, detailed provisions to govern embryo research are unworkable within the international context; individual nations are much better placed to regulate such practices in light of their local cultural and regional conditions.

The authors of the declarations have addressed these criticisms by arguing that while they may include principles that occasionally seem inconsistent, ethical decision-making in practice frequently requires deliberation and the weighing of relevant principles. Indeed, by the nature of their goals, the declarations are inherently based on balancing contradictions; they aim to protect individual rights and liberties while enshrining the role of science in helping civilization progress, as well as to remind the international community of its duty of solidarity toward underdeveloped countries and vulnerable groups that face exclusion from the benefits of biomedical progress [5]. Therefore, to advance decision-making, these principles must be understood as complementary and interrelated.

From our perspective, achieving international consensus requires that at least the core elements of the declarations or reports should not be easily changed over time, regardless of the changes in technology and society. The dialectics of continuity and change reflect moral consensus/conflict. More specific implications of the core values are more dynamic over time, although their compatibility with fundamental principles should be preserved [49]. This would be in line with a broader version of “specified principlism” put forward in an attempt to overcome the problem of assigning priorities to conflicting ethical principles [50]. However, “specified principlism” has been used for clinical case resolution, whereas our approach locates the rebalancing of values in the broader context of technological and social changes.

This study aimed to present a descriptive, sociologically oriented analysis of the transformative social valuation of the human genome implied by the UNESCO declarations and the IBC report, focusing on the shifting balance between collectivism and individualism, where different principles are emphasized. Genetics and genomics have gradually shifted from a science based on broad similarities to a study of differences among groups, communities, and populations. The public increasingly encounters genomic testing and screening, not as patients or individuals with a family history that makes them candidates for testing, but as members of specifically targeted groups, such as parents of newborns, biobank donors, and users of expanded population screening. Public communication on this will benefit from early, systematic education, beginning with youth. This will require new terminological frames for addressing such participation at both the individual and community levels, which will affect human decision-making. While the changing valuations in the declarations follow technological developments and social needs, reflecting the diversity of human voices is also critical. Present and future scientific discoveries are likely to reveal even greater fragmentation based on geography, race, and gender, allowing the genetic classification of not only health and disease but also personal traits, such as intelligence. As a result, the risk of discrimination may increase. Therefore, almost 10 years after the IBC report, it is time to seriously consider future directions and policymaking in light of the UNESCO declarations.

## Data Availability

Data sharing does not apply to this article, as no datasets were generated or analyzed during the current study.
